# Dance Is More Than Meets the Eye—How Can Dance Performance Be Made Accessible for a Non-sighted Audience?

**DOI:** 10.3389/fpsyg.2021.643848

**Published:** 2021-04-16

**Authors:** Bettina Bläsing, Esther Zimmermann

**Affiliations:** ^1^Fakultät Rehabilitationswissenschaften, Musik und Bewegung in Rehabilitation und Pädagogik bei Behinderung, Technische Universität Dortmund, Dortmund, Germany; ^2^Fakultät für Psychologie und Sportwissenschaft, Neurokognition und Bewegung—Biomechnanik, Universität Bielefeld, Bielefeld, Germany; ^3^Institut für Lehrer*innenbildung, Inklusive Pädagogik, Universität Wien, Wien, Austria

**Keywords:** dance, visual impairment, blindness, audio description, movement sonification, multimodal perception

## Abstract

Dance is regarded as visual art form by common arts and science perspectives. Definitions of dance as means of communication agree that its message is conveyed by the dancer/choreographer via the human body for the observer, leaving no doubt that dance is performed to be watched. Brain activation elicited by the visual perception of dance has also become a topic of interest in cognitive neuroscience, with regards to action observation in the context of learning, expertise and aesthetics. The view that the aesthetic experience of dance is primarily a visual one is still shared by many artists and cultural institutions, yet there is growing interest in making dance performances accessible for individuals with visual impairment / blindness. Means of supporting the non-visual experience of dance include verbal (audio description), auditive (choreographed body sounds, movement sonification), and haptic (touch tour) techniques, applied for different purposes by artists and researchers, with three main objectives: to strengthen the cultural participation of a non-sighted audience in the cultural and aesthetic experience of dance; to expand the scope of dance as an artistic research laboratory toward novel ways of perceiving what dance can convey; and to inspire new lines of (neuro-cognitive) research beyond watching dance. Reviewing literature from different disciplines and drawing on the personal experience of an inclusive performance of Simon Mayer's “Sons of Sissy,” we argue that a non-exclusively visual approach can be enriching and promising for all three perspectives and conclude by proposing hypotheses for multidisciplinary lines of research.

## Introduction

Dance has been defined and characterized in many different ways and from the perspectives of different disciplines. According to recent perspectives in this discourse, dance can be characterized as an expressive movement performed in the service of communication (Leach, 2014). Orgs et al. (2016) explicate: “We conceptualize the performing art of dance as a human socio-cultural activity where one individual moves, and another watches.” In their essay on the aesthetic communication in dance, they leave no doubt that the modality of the act of communication that takes place between the dancer and/or the choreographer as sender and the spectator as receiver is primarily visual. The medium of this act of communication is the human body in motion that conveys a visual message with emotional (and other) content that is then decoded by the receiver, on the basis of his/her visual and motor action experience (e.g., Calvo-Merino et al., 2010; Orgs et al., 2018). Aesthetic evaluation (enjoyment) of the perceived movement depends on the observer's expertise (Kirsch et al., 2015; Orgs et al., 2018), the variability and predictability of the movement kinematics and dynamics (Orlandi et al., 2020b) as well as the temporal coordination between dancers (Vicary et al., 2017). These perspectives support a common notion in science as well as the arts, leading to the conclusion that the aesthetic experience of dance is mainly a visual one: dance is brought on stage in order to be watched, and even though this visual experience of moving bodies can elicit a variety of multimodal perceptions (Boucher, 2004), it is always (conceptualized to be) visual in the first place. But does this mean that dance as an art form and aesthetic experience is by definition only accessible to a sighted audience?

Obviously, there is increasing interest on the part of dance professionals in giving people with visual impairments access to dance productions that have been created primarily for a sighted audience (e.g., Brand et al., 2019; Whittenburg, 2019). While audio description and touch tours have proved to be successful in facilitating the participation of visually impaired and blind audiences in cultural events such as theater productions (Ferziger et al., 2020), this might, at first sight, be a more difficult encounter for dance than for other performing arts domains. As dancer and choreographer Naomi Brand puts it: “If you take the visual away from dance, what is left? What are we performing when we aren't being watched?” (Brand et al., 2019). According to Christensen and Calvo-Merino (2013), three components play a decisive role in the perception of dance: the body, movement perception and emotion. The body is the element that carries the artist's message, turning movement into dance. The perceptual experience is a cognitive process that takes place when viewing human (dance) movements. The third aspect assumes that emotions are primarily communicated via facial expression, static body postures and movements. Other factors that contribute to the dance experience include costumes, decorations, stage design, light, colors, symmetry, synchronization, shapes and choreographic structures. These parameters are almost exclusively conceptualized from the viewer's perspective, based on the visual perception of dance. It might indeed be challenging to convey to a person with blindness the specific quality and perfection of a dance performance, especially if that person has no visual experience at all, and most dance professionals arguably know little about how people with congenital blindness experience the world. Nevertheless, those born blind have access to essential aspects that make up dance, including the human body, own and others' movements, social interaction, emotions, space, geometry, sound, rhythm. All these aspects are accessible in other ways than through sight alone, even though they may present themselves differently if they are approached in a way other than visual.

The central message of this article is to show that a non-exclusively visual approach has the potential to enable any audience, sighted or not, to experience dance in an enriching way. It is primarily inspired by the question how dance can be made accessible and brought to life for audience members who are blind or visually impaired. The case study of an inclusive performance of Simon Mayer's “Sons of Sissy” experienced by one of the (sighted) authors is used here as an example and test case for this approach. Equally important is the idea that (especially contemporary) dance can be a laboratory in its own right in which dance artists are the researchers who question, explore and expand the boundaries of human perception and cultural concepts (e.g., Pakes, 2009; Gilette and Pietsch, 2016). This applies in particular to inclusive dance settings in which dancers with different bodily and sensory conditions participate and interact (Østern, 2009; Quinten and Bilitza, 2021). Shifting the sensory mode of communication away from the almost exclusive visual represents an exciting perspective in this regard. Third, building up on these perspectives, we propose hypotheses for future studies, hoping to inspire research that acknowledges scientific and artistic perspectives alike.

## Experiencing Dance With all Senses

In the following, we will address different modalities in which dance can be accessed, from the visual (section Watching dance, and enjoying it) over the audible to the tangible (section Action perception coupling beyond vision). Audible access to dance will be regarded in detail, with three different aspects: natural sounds produced by the dancers (section Dance made audible by means of choreography), technically produced sound resulting from movement sonification (section Dance made audible by means of technology), and audio description (section Putting dance into words: challenges for audio description), followed by tangible access via touch tours (section Dance performance made tangible). In typical “real world” scenarios, none of these modalities (except, to some extent, the visual) would be used exclusively to provide access to dance performance, supporting the notion that dance is a multimodal activity, for the producer as well as for the perceiver. How the different access modalities are combined and integrated will be the topic of subsequent sections.

### Watching Dance, and Enjoying It

If one regards dance as a communicative process (Orgs et al., 2016), the modality of this communication is primarily visual, its medium is the body, which is above all to be seen—this is where dance differs from music with which it shares common roots. From an anthropological perspective, dance and music have formed a unit over long phases of their evolutionary history (and in many cultures until today), both are rhythmically based behaviors that are common in almost all human cultures and play important roles in different social contexts and functions (Trehub et al., 2015; Fitch, 2016; Fink et al., in press). The separation of dance and music in Western cultures (Trehub et al., 2015) thus entails an attribution of the primary sensory modalities, assigning music to the auditory and dance to the visual domain. Nevertheless, dance offers much more than just a visual experience. According to Boucher (2004), the aesthetic experience evoked by the visual experience of dance is a multisensory one based primarily on kinaesthetic synesthesia. Aesthetic pleasure is created by the fusion of different perceptions, which form a “shape” and thus lead to an increased embodiment and inner simulation of movement, a feeling of physical enthusiasm. From the side of dance practice, these finding are supported by the literature on somatic practices and their role in increasing dancers' awareness of their own body to enhance movement creativity and expression (Eddy, 2009; Coogan, 2016). This perspective prevails in the multidisciplinary project Watching Dance that focused on the kinaesthetic empathy of the audience, their physical experience of the movements of the dancers (see Reason and Reynolds, 2010; Jola et al., 2012). The mental simulation of observed movement is backed up by scientific findings that show that the execution, observation and mental simulation of movement are largely based on the activation of the same brain regions and that their degree of activation while watching depends on the observer's own movement experience (Calvo-Merino et al., 2005, 2006; Cross et al., 2006). Parietal areas belonging to this action observation network (AON, Grafton and Hamilton, 2007) are particularly activated during the observation of dance movements judged as both aesthetically attractive and difficult to perform (Cross et al., 2011). Further support comes from studies of motor imagery in dance, showing that brain activation and imagery quality are modulated by dance expertise (Fink et al., 2009; Orlandi et al., 2020a).

Growing interest in aesthetics has shifted the focus of neurocognitive research toward interactions between aesthetic evaluation, visual familiarity and motor experience (Cross and Ticini, 2012; Christensen and Calvo-Merino, 2013). Kirsch et al. (2015) found that physical practice as well as repeated watching of a dance phrase increased its perceived aesthetic value and positive emotional reactions in response to watching it, and interpreted this result in terms of increased perceptual fluency that goes beyond the one underlying the mere exposure effect (Orgs et al., 2013). FMRI results revealed that after the physical training, brain regions involved in the aesthetic evaluation shifted from subcortical dopaminergic reward circuits to the superior temporal sulcus (STS) that plays a crucial role in the perception of human body movement (Blake and Shiffrar, 2007; Vangeneugden et al., 2014), bodily expression of emotions (Grèzes et al., 2013) and multisensory integration in the context of musical rhythm perception and movement coordination (Chen et al., 2009). Kirsch et al. (2015) concluded: “the more modalities through which an observer has experienced a movement, the more enjoyment the observer derives from watching that movement.” In a comprehensive review article, Kirsch et al. (2016) discuss the complementary roles of visuomotor, sensorimotor, and reward systems and their interactions in the perception and aesthetic evaluation of human body movement. They relate findings from numerous studies in neuroaesthetics to the embodied simulation account of aesthetics (Freedberg and Gallese, 2007), a framework that explains aesthetic experiences in the arts on the basis of action simulation and bodily sensations elicited by the perceived piece of art. Kirsch et al. (2016) propose that expanding the focus of neuroaesthetics research to include dance and human body movement in general could lead to a more holistic understanding of aesthetics. At this point it could be added that, given the prominent role of brain areas supporting multimodal integration and supramodal representations (Ricciardi and Pietrini, 2011), not exclusively focusing on visual perception could be another promising step into this direction.

### Action Perception Coupling Beyond Vision

While the coupling of visual perception and action has been investigated extensively by many authors interested in dance and movement practice (Yarrow et al., 2009; Bläsing et al., 2012), comparatively few studies exist that focus on action perception coupling for other sensory modalities, including auditory ones (Kennel and Pizzera, 2016). Activation of AON regions has been observed in response to listening to a previously learned piano piece (Lahav et al., 2007) as well as the sounds of steps (Bidet-Caulet et al., 2005). Expertise studies have shown that athletes can recognize sport-specific actions from acoustic information only (Allerdissen et al., 2017; Klein-Soetebier et al., 2020), and that dancers can learn novel movement material from verbal description only (Bläsing et al., 2018). Evidence exists that in individuals who never had any visual experience, areas belonging to the AON are activated by hearing action sounds and while reasoning about mental states of others (Bedny et al., 2009; Ricciardi et al., 2009). In persons with congenital blindness, the auditive perception of actions activates premotor, temporal and parietal cortical areas, and this activation is modulated by action-specific experience (Ricciardi et al., 2009). Rosenblum et al. (2017) support these findings from a neuroscientific perspective, stating that “audition may be performing a much wider range of crossmodal functions than we typically consider. There is evidence that the brain can use audition for functions typically thought to be vision's domain.” These authors ground their review on studies with participants with and without visual impairment, discussing among other topics human echolocation skills. In blind individuals using echolocation, visual cortex areas are involved in the identification of objects based on shape recognition or surface exploration (Thaler et al., 2011; Arnott et al., 2013), including Braille reading (Cheung et al., 2009). Ricciardi and Pietrini (2011) provide evidence that cortical areas belonging to both visual pathways do not exclusively process visual information, and that they can be recruited by other sensory systems through crossmodal plasticity in the absence of vision. They propose that object recognition and concept formation rely on supramodal cortical areas that do not require visual input nor visual imagery to function or develop, and that can be recruited in a top-down manner by higher cognitive functions, including memory processes or action planning.

These perspectives are supported by research showing that mental imagery can arise from other than visual modalities, in individuals with or without visual impairment, including those who were born blind (Arditi et al., 1988; Renzi et al., 2013; Holsanova, 2016). People with congenital blindness, however, experience mental imagery in ways that differ from those experienced by sighted people, with more spatial, tactile and kinaesthetic content and in closer connection to semantic knowledge (Röder and Rösler, 2004; Cattaneo and Vecchi, 2011). Spatial imagery in individuals with visual impairment preferably applies an egocentric frame of reference, with landmarks playing a major role for orientation and navigation (Noordzij et al., 2007). Studies with skilled blind football players revealed that spatial representations and navigation skills in individuals with visual impairment are sensitive to sport-specific training (Velten et al., 2014, 2016). Ricciardi and Pietrini (2011) point out that spatial discrimination and navigation skills in individuals with visual impairment are comparable to those in sighted, and that this also applies to cognitive domains that rely indirectly on spatial representations, like number processing. Taken together, these findings provide evidence that human motor action can be explored in other ways than through vision. In the following, we will address different ways how dance can be perceived in non-visual ways, including sound, spoken language, haptic exploration and the experience of one's own movement.

### Dance Made Audible by Means of Choreography

Dance, and human motion in general, is not only visible, it can also be heard. While in classical ballet productions as well as in numerous modern choreographies the sounds caused by the dancers' footsteps, ground contact, audible breathing or voices take a back seat, individual choreographers use such acoustic signals as dramaturgical means. One example is given by William Forsythe's “breath scores” that include not only audible breath but also noises caused by voice, footsteps, ground contact, body and clothing (Vass-Rhee, 2010). These acoustic events that are generated directly by the dancers' bodies are regarded on the same level as visible movements; in combination, they result in a performative intermodality with which Forsythe experimented intensively in 2003–2005. Vass-Rhee (2010), former dramaturge at The Forsythe Company and cognitive scientist, describes: “Forsythe's intermodal choreographies are dancing music that extends dance into the other, inner spaces of the body, translating the ‘grain’ across the senses of vision and audition. Through performative linkages of kinetic and sonic action, the performers visibly and audibly express the lived experience of their dancing. The body, so often silent in dance performance, is enabled to ‘speak for itself’ and does so on its own terms, asserting its role in the construction of meaning and inverting the hierarchy of language and perceptual experience.” This practice is extremely demanding; besides virtuosity of movement, it requires that Forsythe's dancers also have a virtuosity of perception, the distribution of their attention between inner and outer body spaces and between their own body movements and noises and those of others (Vass-Rhee, 2010).

The question to what extent audiences enjoy hearing the sounds produced by the dancers' moving bodies has been addressed in few studies. Jola et al. (2014) found that spectators' entrainment and evaluation of a dance performance without music depended on their personality traits, with those scoring high in openness showing the highest appreciation for hearing only dancers' natural body sounds instead of any musical score. Howlin et al. (2020) presented a video-recorded dance performance with dancers' natural body sounds and voices either congruent or incongruent with the visible movement, or in silence. In this study, audience members showed the strongest psychophysical reactions and positive ratings for the incongruent condition in which the sound could be interpreted as independent of the dancers' actions, a designed sound score rather than a product of the movement. While sounds produced directly by the dancers might not be interesting or aesthetically appealing for all spectators, they certainly have the potential to convey relevant information about movement dynamics, spatial relations and interactions, in particular in the absence of vision.

### Dance Made Audible by Means of Technology

In addition to the natural sounds produced by the dancers' bodies, movement information can be made accessible in the auditory domain with the help of technologies such as sonification, the acoustic representation of data. Sonification has been used in different arts and science contexts for turning visual into audible information for educational and analytical purposes. Nadri et al. (2019) developed guidelines for turning paintings into sounds that reflected their art style, genre and other selected characteristics. Bowers and Shaw (2014) sonified geological objects and fossils as well as meteorological data to enrich and stimulate visitors' interaction with the collection of a natural history museum They report that “there were very interesting sonic differences between the different materials with, for example, particulate or moisture containing rocks spluttering in a bursty fashion while granite was largely mute,” and reflect that this work stimulated the audience's curiosity and appreciation of the exhibits.

Sonification also offers a wide range of options for making dance audible: movement data that is collected using optical motion capture systems or sensors attached directly to the body can be converted into acoustic signals or sounds (Niewiadomski et al., 2019; Giomi, 2020). What makes this technology particularly attractive is that sonification can be applied in real time, so the acoustic representation of the desired information can be fed back into the ongoing action as a stimulus. Given the multitude of analytical, descriptive or exploratory applications, the challenge lies in choosing the appropriate “translation” of movement data into acoustic signals—what exactly should be sonified, for what purpose, and how? Which parameters of the movement are of interest and how they should sound depends mainly on the functional purpose of the sonification. As an analytical tool, it can filter out or amplify selected features of the movement that are otherwise hard to identify, and can thereby be used to support the learning and training of movement sequences by providing selective acoustic feedback (Großhauser et al., 2012). In an artistic context, sonification can also be used as creative interaction tool to shape movement expression and to produce sound compositions through body movement, which can then be used to stimulate new movement (Landry and Jeon, 2020). Katan (2016) reports on a workshop with three dancers with visual impairment in which the benefits of movement sonification for communication and joint movement were examined. Afterwards, all dancers stated that they enjoyed controlling sound through movement and that they felt encouraged to try new things, however, it did not improve movement comprehension or mutual understanding. Examples of the sonification of dance movement conveying different qualities (lightness, fragility) are provided as video material by Niewiadomski et al. (2019).

### Putting Dance Into Words: Challenges for Audio Description

In many areas, audio descriptions are used as an aid to foster the cultural participation of people with visual impairments and blindness. Museums and galleries offer audio descriptions of static exhibits and works of art, such as paintings or sculptures. As an example, the oil painting “Irises” (1889) by Vincent van Gogh is described as follows: “In this painting, dozens of irises rise up in waves of color, like green and blue flames fanned by a wind that blows them, now flattens them, toward the left. Carried along on thick brushstrokes, it seems as though a wave of motion flows from lower right to upper left, sweeping the shapes of leaves, stems, petals, and blossoms into a wide river of color” (J. Paul Getty Museum, n.d.). Theaters supplement drama productions with audio descriptions in which information about the actors' actions and body language is conveyed (Braun, 2008; Udo and Fels, 2010a; Cavallo, 2015). Audio description has also been used in film productions and television since the 1980's (Kleege, 2016). During pauses in the dialogue, the audio descriptor comments on aspects of the action, gestures, facial impressions, clothing, places, landscape and camera work that are important for understanding the scene. An expressive example of an audio description for film is given by Bardini (2017) for the black-and-white short movie “Nuit blanche” by Manoukian (2006). The author illustrates how the key scene of the movie can be described in different ways, depending on audio description style that can be cinematic (“When they are few inches from each other, they close their eyes and bring their lips closer as they are about to kiss. The frame focuses on their lips, which nearly touch. It passes beyond them and returns to reality, at normal speed”) or narrative (“In front of each other, they look at each other's lips and close their eyes as they are about to kiss. Suddenly, everything goes back to reality”).

Forty years later, audio description is now also used more frequently in dance productions. Dance, however, provides specific challenges for audio description—unlike works of the fine arts, dance works are fleeting and constantly in motion; unlike film and drama, they are often entirely non-verbal, and their content is often emotional, associative and metaphorical, rather than semantic. Dance, like music, is not communicative in the sense of sharing declarative information, but feelings, experiences and social cues (Trehub, 2003). A complete description of everything that happens on stage in a fully equipped ballet is hardly possible: in addition to the complex movements and different qualities of multiple dancers, their interactions and the spatial composition, the costumes, the set and the props would have to be described in real time and without interfering with the music. The main problem for audio description arises from the temporal structure of dance as a dynamic, ephemeral art—what the visual system can evaluate in a highly efficient manner in milliseconds would need at least several minutes to be verbally described in adequate detail. Furthermore, certain details such as facial expressions, hand gestures and body language are often too elusive and subtle to be described adequately in words, yet they are of central importance in non-verbal communication. While in drama and film many hand gestures can be identified as emblematic, linguistic or symbolic and many facial expressions represent emotions (Mazur, 2014), this is not necessarily the case in dance, where bodily and facial expression, like body movement, are often applied in explorative ways.

One of the major challenges in dance audio description is the necessity to filter information rigorously and select what is relevant and what is not, and which level of detail is appropriate. Here, a conflict arises from general guidelines for audio description, which propose using factual, objective and non-judgmental language that informs what can be seen without interpreting it (Braun, 2008; Kleege, 2016). It remains questionable whether this is appropriate for dance. Asking how movement can be translated into words in a meaningful way, Margolies (2015) provides an example from the dance piece *(i)land* by Marc Brew Company: “She steps slowly onto the island, kneads the sand with her toes. She twists from side to side, her gaze fixed on the sand. She pushes her toes through it, lifts one foot, balances, lets the sand trickle off her toes. She spirals, her head turns from side to side, her arms follow, still moving with the sea.” Margolies (2015) demands that audio descriptors for dance should be trained in the same way as translators of poetry, whose task it is to convey not only concrete semantic content, but also aesthetic aspects of language, its sound and rhythm and associative images that are generated while reading. The author explains: “Like a translator, a describer makes decisions about the intended meaning of the original and the frame of reference of the audience. The process always involves some loss, but the compensations and substitutions can sometimes be felicitous, metaphorical. At best, a description is a negotiation between members of the creative team, but it will always be partial, reflecting the viewpoint, experience and limitations of the describer” (Margolies, 2015).

The perspective expressed by Margolies (2015) is supported by results of a study on audio description for film. Bardini (2017) compared three different description styles (denotative, cinematic and narrative; see excerpts at the beginning of this section) for a short film in a between-groups setup with 39 participants. She found that among the 15 participants who showed the most emotional responses, immersion and enjoyment, nine had heard the narrative audio description, five had heard the cinematic and only one had heard the denotative version. Walczak and Fryer (2017) found that for a naturalistic drama, “creative” audio description that includes cinematic cues and strong, emotive language was well accepted by the audience, and preferred to standard audio description in particular by male audience members. Going a step further, Cavallo (2015) describes theatrical practices in which the audio description is provided by the performer from a first-person perspective and intertwined with performative language (ekphrasis, storytelling), it thereby becomes a part of the performance itself instead of “hiding behind headsets”. Cavallo (2015) argues that this practice has a potential for “strengthening the already inherent link between word and image, and allowing description to act as a ‘representation of representation’.” Another aspect that should be considered with respect to the non-declarative nature of dance as means of communication is the potential of the human voice to convey via prosodic cues, intonation and emphasis what is not directly expressed in words. Colmenero and Gallego (2020) emphasize that vocal expression and modulation, dramatic pauses or voice breaks can elicit a sense of subjectivity, which can be desirable in audio description to indicate for examples metaphors or irony. For dance, such cues should be marked in the audio describer's script in collaboration with the choreographer, to make sure that the intonation and vocal cues reflect and support the choreographic message.

### Dance Performance Made Tangible

In addition to the audio description provided during the performance, some theater productions offer “touch tours” that take place before the performance for audience members with (or without) visual impairment (Udo and Fels, 2010b). The participants have the opportunity to enter the stage area, touch objects, props and costumes. They might also meet the dancers or actors and be given the chance to talk to them, hear their voices, and to shake hands with them. For few dance productions, such preparatory tours include the offer to try out and learn simple sequences of movements from the production itself through description and haptic guidance. If these movements are then named in a catchy and pictorial way (Fryer, 2009), they can be easily recognized in the audio description and, thanks to the previously made sensory-motor movement experience, act as concrete points of orientation in the mental imagination of the choreography described. While a touch tour can show what the room, the set, costumes and props and individual movements feel like, it conveys little of the impressions that the sighted audience experiences during the performance. The “on-stage” perspective, which is actually that of the dancer, can be interesting and enriching, but it is, after all, limited in providing audience members with visual impairment an audience perspective that is aesthetically comparable to that of sighted spectators. Another means of making dance performance tangible is to add three-dimensional pictures of body postures and scenes from the performance in an exhibition that accompanies the performance, for audience members to explore haptically. The extent to which earlier visual experience is useful or necessary for the interpretation of such images is under debate, however, even audience members with congenital blindness can extract useful information via tactile picture perception, especially if additional information or explanation is provided (Heller, 2002).

## A Case Report: Simon Mayer's “Sons of Sissy”

The question how a dance performance can be made accessible for audience members with visual impairment will be examined using the example of Simon Mayer's “Sons of Sissy,” a choreography between contemporary and folk dance. In July 2016, the piece was performed as part of the project “The Humane Body—Ways Of Seeing Dance” (www.thehumanebody.eu/) during the ImpulsTanz International Dance Festival in Vienna (Impulstanz, 2016). “Sons of Sissy” premiered in 2015 (Ploebst, 2016) and was presented in 2016 in the Odeon Theater in Vienna with a version that included a touch tour and audio description, as well as an exchange of experiences between the artists and their audience after the performance. The third section of this article focuses on this performance experienced by one of the authors (EZ) from the perspective of a sighted audience member. More detailed information about the piece and the performance and a personal protocol of EZ's impressions of the performance are provided in the Supplementary Material.

### Experiencing Dance *via* Sound

Commenting on his collaboration with “The Humane Body—Ways of Seeing Dance” on his piece “Sons of Sissy,” Simon Mayer states: “Feeling or knowing blind people in the audience expands our consciousness. With regard to the sound that we create and perform live, but also because as a performer you are not used to having people sitting in the front row who do not ‘look at’ you, but listen to you” (Simon Mayer, personal conversation). Simon Mayer expressed that being aware of the blind audience members reminded him of what the rehearsal was originally about: sound, music and a visual audio composition; and thus he felt “audio-sensitized” again. He regarded the sound of the piece as very important right from the start. Sound generally plays a major role in all his works, he therefore even records albums from the sounds and music of the pieces if possible. Imagining what it would be like to be blind and just hear the piece makes the “audio component really strong and compact.” He therefore often uses dance movements that generate sound, such as stamping, slapping or clapping, and pays attention to technical tools such as the right microphones to amplify the sounds. The dramaturgy of music and noise is a great means for the choreographer to make movement audible and interpretable. Even with audio description, there is still space for interpretation, which is important to the choreographer. Mayer tries to capture the message of his pieces aurally, so that one “can feel the essence of the concept” from listening to it, and hopes to refine the music and sound dramaturgy to such an extent that audio description is no longer necessary. Nevertheless, he regards audio description as part of the choreographic composition and favors the idea to offer various audio commentaries, with and without interpretation: “It is easy to imagine that if you have time to work on it, audio commentaries could do justice to the piece with all of its intentions.”

### The Audio Description

When asked how words can do justice to movements, Valerie Castan, herself a dancer and performer and Europe's only specialist in audio description in the field of dance (cf. Impulstanz, 2016), answered that she tries to be as factual and concrete as possible in the description. In Castan's opinion, the description should be interpreted as little as possible, but, as with the translation of a book, interpretation can never be completely avoided. Interpretation that enters the audio description should be discussed with the choreographer and dancers beforehand. The text for the audio description is fixed in advance, but improvisations and changes in the choreography can occur and have to be dealt with. It is important to leave pauses for music, noises and rhythms that can be perceived immediately. For describing bodies and movements, metaphors should be used whenever possible, and technical terms and specific dance vocabulary should be avoided, so that the listeners can follow without prior knowledge. Castan finds it particularly difficult to translate the humor that certain movements produce: “Whatever gets the most laughs is when the dancers are completely naked and are jumping. And since they are jumping naked, their penises also jump up and down. It's pretty silly and the audience laughs every time. … So I wait until the audience laughs and then say: everyone's penis jumps up and down” (Valerie Castan, personal conversation).

### The Touch Tour

Before the performance started, both visually impaired and sighted (blindfolded) audience members had the opportunity to take part in a touch tour. Castan considers the touch tour to be very useful for the audience to create a kind of “kinaesthetic empathy” before experiencing the performance via the audio description. Holding tight to each others' shoulders, participants formed a “snake” and moved around to explore the spatial characteristics of the stage while listening to a spoken description. The participants were allowed to touch the walls and props, such as chairs and benches; whips, tinhorns and hanging cowbells were passed around to be explored haptically. Then they walked together in a circle, pounding right and left in the same rhythm, while the choreographer was standing in the middle giving instructions. Participants were asked to stop and tap each other alternatingly on the thighs, which would then turn into the “Schuhplattler” (a traditional dance style from the Alps region in which the dancers stomp and clap their hands on their thighs, knees and shoes). At the end, the choreographer yodeled a tune, which the circle then repeated.

### Audience Reflections

According to visually impaired audience members, the touch tour was perceived as a good and necessary introduction, but it could have been more extended. Noises made by the materials and people walking or clapping, the dimensions of distances on the stage could be recognized later during the performance thanks to this introduction. One audience member, however, criticized the fact that one was only in the perspective of the dancers, he lacked the reference to the grandstand, from where one should ultimately perceive the piece later. During the performance, the music was experienced as very impressive, it was difficult for the listeners to imagine the dancers playing instruments and dance at the same time. The sounds of the instruments gave a good impression of where the dancers were located on stage, but sometimes the background noise, especially when whips and bells were used, was too loud. Descriptions of movements and interactions were sometimes hard to follow. Crucially, sometimes it was unclear why the sighted audience members laughed because the audio description did not respond to funny aspects of the performance (e.g., related to the nudity of the dancers) or reported only afterwards why people had laughed. Individual audience members mentioned that the audio description included too much detail and did not leave enough pauses for creating one's own ideas, or that the piece was easy to follow even without audio description.

### Author's Reflections

According to Christensen and Calvo-Merino (2013), dance depends on culture, tradition, the respective applicable morals and respected aesthetics. Dance is characterized by physical movement, typically performed with music, it serves as a form of communicative expression, and enables social interaction or takes place in a spiritual or performative context. The body, its movement and expression of emotion are the major elements that determine how dance is perceived, other contributing factors are costumes, decorations, props, stage design, light and colors, symmetry, synchronization, form, choreographic structures and the functional sense of the events. In the following, I (EZ) will examine to what extent these aspects of dance are represented in my non-visual experience of the performance of “Sons of Sissy” presented here as case study.

The first aspect, the ability to experience culture, the tradition in which the dance takes place, is provided by the audio description that in this case describes the folk costumes. A more precise idea could be formed if the clothing could be haptically explored in the touch tour. Audio description can also be used to communicate whether the dancers' demeanor and movements correspond to current moral and aesthetic views. The mention of nudity, which in the context of a traditional folk dance represents a break with the conventional plays a decisive role in the interpretation of the piece. The aspect of dance being performed to a musical rhythm can directly be experienced by a person with visual impairment, due to the presence of sounds, noises and live music, even without audio description. The spiritual character of dance was supported in “Sons of Sissy” by the pounding rhythms that created a ritualized feeling and the smell of incense, the performative character was created by the typical stage environment. The functional purpose of dance does not require audio description either; in this case, the piece was presented for entertainment.

The extent to which dance represents a form of emotional expression and social interaction depends strongly on the audio description. The choice of words can steer in a certain direction and thus anticipate an expression, or it can use rather neutral phrasing and thus leave room for the listener's own interpretation. In the performance of “Sons of Sissy,” the live audio description (provided by Bernd Kainz, who had been responsible for the Austrian Broadcasting Corporation's audio description on the 2016 Summer Olympic Games) contained both open and more focused descriptions, in a rather factual language (e.g., “S. stops playing and lets the violin sink down”; “the lighting mood changes”; “M. jumps the hardest”; “P. stands on the missing side of the circle”; “they walk in a radius of about four meters”). It remains questionable whether metaphorical phrases such as “as if he were imitating a bird” could give a more appropriate idea of the actual movement. Furthermore, it has to be asked how important it is to have a supposedly “correct” idea of a movement, and which criteria the “correctness” of a movement description would depend on. It has to be born in mind that the communication of emotions strongly depends on even subtle facets of facial expression, as well as on body posture and movement quality and their respective interpretations. Taking all these aspects into account would be a mammoth task for the audio descriptor that could hardly be achieved in real time, considering in particular that vision is never free from learned social factors that determine in a top-down manner what is selected and what is filtered out (Mraczny, 2012). The audio description thus has to take on the role of camera guidance, deciding for the audience where to focus attention (An extended and more detailed personal reflection can be found in the Supplementary Material).

## Dance as a Non-visual Multi-Sensory Experience

In the previous sections, we have focused on approaches and techniques that are used to make the experience of dance performance accessible to a non-sighted audience. The question to what extent these approaches are successful in reaching persons with visual impairment and making them enjoy and appreciate the aesthetic experience provided by dance, has not yet been given much space in this article. In the following, two scholars' reports about their own non-visual dance experiences will be presented, one of them a trained dancer, the other one a literature theorist with no specific interest in dance.

Literary scholar Georgina Kleege lost her eyesight during childhood, but still completed her training as a professional dancer at the Martha Graham School, New York. Kleege (2014) sees audio description as generally problematic, to her the dictum that the description must not be subjective and emotional without personal impressions and evaluations makes little sense. She doubts that words describing a work of art can conjure up an image in the mind's eye of a blind person, which can then be the subject of independent personal aesthetic evaluation—she even doubts having anything like a “mind's eye” herself (Kleege, 2014). Notably, this does not apply to all individuals with visual impairment, as revealed by research on mental imagery in persons with congenitally blindness (Arditi et al., 1988; Renzi et al., 2013; Holsanova, 2016; see also section Action perception coupling beyond vision). Kleege (2014) compares audio description of dance with dance criticism, which is supposed to convey personal impressions and evaluations as well as a competent professional classification. Ultimately, she asks whether language in general can be a suitable medium for conveying dance to non-sighted people: “If words are the problem, can dance be experienced through other modalities, allowing the blind audience member to have an unmediated experience?” To answer this question, the author reports about a workshop by choreographer Victoria Marks, in which touch was addressed as mediating sensory modality. The participants were involved in short, repetitive movement sequences through touch, with changing dance partners. Kleege (2014) describes that as a participant, she was intensely aware of the different qualities of the changing dance partners and that learning the repetitive movements created a positive, joyful tension that reminded her of her dance studies. She adds, however, that not all participants experienced the physical closeness and the situation of being touched and moved as pleasant. In general, she reflects, the situation of touching and moving together aroused strongly emotionally tinged memories and was thus “touching” in several ways.

Piet Devos, who is also blind since childhood, comments on two dance productions he experienced: “NEWS”, a solo without music choreographed by Deboray Hay and danced by Bettina Neuhaus in 2007, and Eline van Ark's “De Onzichtbare Danser” (2013–2015), which was choreographed exclusively for a non-sighted audience (cf. Devos, 2018). The author perceived both dance productions as intense multi-sensory experiences that did not only have a significant influence on his thinking about dance, but also about the possible role of a non-sighted audience as addressees and source of choreographic ideas (Devos, 2018). During the presentation of NEWS, Devos reports, the physical experience of sound (the dancer's footsteps, the rustling of clothes, breathing, whispers and bird-like screams), space, and the presence and movement of the dancer formed into an intense aesthetic experience of being involved. These immediate sensory perceptions aroused strong emotions and evoked immediate physical reactions such as laughter, shock or empathy for supposed pain in the author, and finally the wish to reach out and touch the dancer.

Eline Van Ark went one step further in developing a non-visual choreography. During the first rehearsals for “De Onzichtbare Danser” (2013), Devos (2018) reports, she invited blind guests as “listening experts” to find out which of the dancer's actions they would find acoustically appealing. The rehearsal audience consisted of people with and without visual impairment, with the sighted experiencing the rehearsals blindfolded. Van Ark thus used visual impairment as “positive resource” for the choreographic process, as a source of special experience and knowledge of a sensory practice that is otherwise mostly neglected in dance. Repetition of acoustic patterns played a special role in this non-visual choreography, allowing the audience to recognize what they had already heard and to fill it with meaning. Devos (2018) was particularly impressed by the development of a common multi-sensory, non-visual aesthetics within the rehearsal audience. He sums up: “Whether through the auditory exploration of space and presence or the haptic appropriation of another's movement into one's own body, blindness brings along an intensely affective, sensory plenitude to the dance theater, which is at odds with the formalism and distanced judgment typical of the more conventional, predominantly visual approach to dance. The result of such promising experiments within contemporary dance is the emergence of a more inclusive aesthetics in which the creative potential of sensory differences is no longer suppressed, but ultimately acknowledged.”

## Discussion

In this article, we have initially asked the question if dance is by definition a visual art form and thereby only accessible to a sighted audience; as expressed by Brand et al. (2019): “If you take the visual away from dance, what is left?” Looking at the many facets of dance and what is written about them, there are many reasons to assume that this should be the case; however, central aspects like the human body, movement, social interaction and communication, emotional expression, timing and rhythm, entrainment and coordination, are neither exclusively nor predominantly visual in nature, but multimodal. This means that, if the visual is taken away, dance should still provide a rich aesthetic experience, given that the communication of what is relevant to dance can use more channels than just the visual. To account for this, different ways of enhancing the non-visual qualities of dance or translating dance into other modalities have been applied, several of which have been addressed in this article. Reflecting upon the personal experience of an inclusive performance with touch tour and audio description of Simon Mayer's “Sons of Sissy” as example and test case, we discuss the chances and challenges dance provides in particular for audio description. The audience's and author's reflections showed that even though the experience was positive and enriching for sighted as well as non-sighted audience members, the task of making dance performance accessible for persons with visual impairment still offers many open questions for artists and scientists alike.

In the previous section, we have presented perspectives brought forward by Piet Devos and Georgina Kleege, two scholars with substantial visual impairment, one of them with a professional background in dance (Kleege, 2014; Devos, 2018). Common to both perspectives is the notion that the authors' preferred ways of experiencing dance would be rather direct, multimodal and physical than mediated through a translation of the viewer's perspective. Both authors refer to choreographies that had been created for or even in collaboration with a non-sighted audience, using the sounds evoked by the dancers' movement and body, voice, touch and even the audiences' own guided movement in order to create a rich and immersive aesthetic experience. Both authors agree that their respective experiences were positive, immersive and emotional, and that they felt motivated to move and interact. These reports, along with other examples of interactive multimodal choreography and production (e.g., Whitfield and Fels, 2013; Fryer, 2018; Brand et al., 2019) suggest that it might be a good idea in general to think dance as multimodal and potentially non-visual art form from the beginning of its creation, in order to address a non-sighted as well as a sighted audience. While this approach could be of fundamental relevance for the cultural participation of persons with visual impairment, it could also inspire exciting interactive experiences for sighted spectators, like René Reith's choreographic project [IN]SIGHT (Reith, n.d.).

Besides such interactive and multimodal approaches to choreography, a huge body of dance works exists that has been and will be produced primarily to be watched, aiming at a mainly visual aesthetics. Making these works (including a large body of historically relevant archived material) accessible for a non-sighted audience will have to rely on techniques that have been addressed previously in this article, including audio description, sonification and touch tour. In order to further develop and improve these tools and techniques, we would like to encourage scientists and (dance) artists to engage in research that helps to shed light on two questions: (1) how can dance be made accessible for persons with visual impairment, including (congenital) blindness, and (2) how can dance “function” in a broader sense without the (pre-)dominant visual quality? While the first question is motivated by discourses in disability studies and cultural participation (Whatley, 2007; Quinten and Bilitza, 2021), the second question could be of interest for dance as an art form that seeks to explore and expand the boundaries of human perception and culture. It should also be relevant for cognitive science, as it touches fundamental disciplinary topics such as multimodal representations, the coupling of action and perception, the generation of concepts, the grounding of language, and the segmentation and encoding of events (Holsanova, 2016). In the following, we will first elaborate on findings from neurocognitive research that could inform and inspire the multidisciplinary search for non-visual access to dance (section Neurocognitive perspectives in multimodal dance perception), before we outline ideas for three fields of future studies: audio description for dance, dance and movement sonification, and multimodal, interactive choreography.

### Neurocognitive Perspectives in Multimodal Dance Perception

In recent years, research in neuroscience and cognitive psychology has focused on topics that are highly relevant for understanding social cognition and interaction, and dance has not only become a valuable source of inspiration for this research, but has also contributed to methodological approaches. Several studies in this field provide empirical evidence that synchronous (rhythmic) movement can have strong positive effects on emotional and social factors and even reduce pain perception (Tarr et al., 2015, 2016; Lang et al., 2017; for prosocial and antisocial affects of behavioral synchronization, see Gelfand et al., 2020). Personal affiliation within groups moving in synchrony arises from distributed coordination between individuals moving in synchronized dyads, rather than unitary synchrony of individuals moving in unison to a common rhythm (Von Zimmermann et al., 2018). Vicary et al. (2017) showed that the aesthetic evaluation of observed movements is influenced by the synchrony and temporal coordination between the dancers. In their study, a specifically created choreography built from simple moves (e.g., walking, running, and arm swinging) that manipulated synchrony among the 10 dancers in the absence of an external rhythmic signal was live performed. Performers' acceleration and observers' heart rates were measured via wrist sensors, and enjoyment ratings were collected from a subgroup of audience members throughout the performance. Results showed that synchrony among the dancers was a stronger predictor of audience engagement and enjoyment than dancers' motion and acceleration, supporting the prominent role of collective movement dynamics in communicating social information (Orgs et al., 2016; Fink et al., in press). In a study on audience reactions to Myriam Gourfink's slow choreography “Souterrain” (2014), Bachrach et al. (2015) showed that spectators' attention to their own and the dancers' breathing was associated with breath synchronization between dancers and the audience. Underestimation of time was correlated with spectators' enjoyment of the piece and engagement with their own breathing and the dancers' muscular activity.

The presented findings corroborate the high emotional and social relevance of movement synchrony and temporal alignment between individuals that affects participants and audiences of the coordinated action. The mechanisms underlying these effects have been linked to rhythmic and social entrainment on different levels (Phillips-Silver et al., 2010; Waterhouse et al., 2014). Interestingly, while vision provides time-locked information about others' (inter-)actions, movement characteristics and spatial arrangements, the basic mechanisms of entrainment do not depend on the visual modality as much as on vestibular, proprioceptive, kinaesthetic and auditive perception (Phillips-Silver and Keller, 2012). To facilitate non-visual access to dance, it could be relevant to investigate how processes of social and rhythmic entrainment and movement synchronization function in the absence of visual input. Even if social and emotional effects are mainly mediated through distributed interpersonal coordination (Von Zimmermann et al., 2018), music and sound can play a major role here beyond providing an external rhythmic signal for unitary synchrony. Music has been shown to influence the perception and aesthetic appraisal of dance (Jola et al., 2014; Bläsing, 2015; Orgs and Howlin, 2020), and it plays a crucial role for movement coordination. Dyads of hip-hop dancers were rated as most attractive when both dancers were moving in synchrony with the music and each other and least attractive when dancers were not in synchrony and only one dancer was dancing with the music (Tang Poy and Woolhouse, 2020). In this study, coordination between the dancers had a stronger influence on attractiveness ratings than dancers' coordination with the music. Christensen et al. (2014) showed that the affective valence of music modulated participants' ratings of ballet movements, and this effect was stronger for sad compared to happy music, while physiological reactions were sensitive to movement arousal rather than affective valance. The authors suggest that the affective experience of dance could be best potentiated if movement arousal and musical tone were in accordance (Christensen et al., 2014).

Taken together, these findings confirm that the perception of dance is influenced by processes of entrainment that do not depend on visual perception but on other factors, including music and sound, bodily cues such as (one's own and others') breathing, and synchrony or temporal coordination of movement. Shifting the audiences' attention toward these factors (e.g., by choreographic means) in addition to the application of audio description and movement sonification could enhance the non-visual perception of dance. Considering respective lines of research, we would like to propose potential research questions and hypotheses for three fields of future studies that arise from individual aspects addressed in the previous sections.

### First Field of Study: Audio Description for Dance

While audio description has recently become a topic of intensive study, dance has not yet been considered by many researchers in this growing field. One reason for this lack of studies might be that audio description for dance is still not as common and well established as it is for other art forms and cultural activities, such as film, drama, fine art, or exhibitions. Another reason might be the range of difficulties and challenges audio descriptors face when working on dance formats, as has been described in previous sections. These open questions could motivate different lines of research asking how the non-verbal, non-declarative, emotional information conveyed by dancers' bodies and movement can be put into words. Investigating perceivers' preferences could be a useful approach, as has been done for other fields of audio description (e.g., Bardini, 2017). Research in different domains including sports, rehabilitation and dance has shown that directing the focus of attention toward external rather than internal movement effects via verbal instructions is beneficial for motor learning (e.g., Wulf and Prinz, 2001; Guss-West and Wulf, 2016). It would be worth investigating if this also applies to the understanding and enjoyment of dance audio description. Hypotheses could propose that participants with (and without) visual impairment prefer dance audio description that uses:

- metaphorical rather than descriptive language for movement;
- global rather than local effects of dancers' actions;
- auditively and haptically grounded metaphors rather than visually grounded metaphors;
- emotional states rather than visual cues of emotional expression (facial expression, gestures);
- egocentric rather than allocentric spatial cues;
- prosodic cues and voice modulations that refer to movement and choreography;
- audible cues and audience reactions as reference.

Hypotheses regarding preferences in audio description could be tested using “silent disco” paradigms in which participants wear headphones and can switch between different channels to choose between descriptions (see Tarr et al., 2016). Furthermore, it could be interesting to develop audio descriptions for recorded material (e.g. from dance archives) interactively in subsequent iterative steps between audio descriptors and non-sighted as well as sighted listeners; this could be useful not only for optimizing audio description styles, but also to develop and improve audio descriptions for archived dance material. Another promising approach could apply segmentation paradigms based on event segmentation theory, which proposes that the on-line segmentation of perceived events determines how episodes are understood and encoded in memory (Zacks et al., 2007; Kurby and Zacks, 2008). Such paradigms have been applied to various activities including dance (Bläsing, 2015; Di Nota et al., 2020). We would expect that comparing the segmentation of dance performance presented either visually or via audio description could provide insights into the ways receivers' perceive and interpret dance on different levels (e.g., “movement” vs. “narrative,” see Holsanova, 2016).

### Second Field of Study: Dance and Movement Sonification

Sonification of dance (and related human movement) has been studied intensively, however, only a small part of this research has been carried out with a non-sighted audience in mind (e.g., Katan, 2016). Investigating this topic systematically could help to gain insights that could establish sonification as a tool for augmenting dance performance. Related questions have recently been addressed in an international project that aimed at developing technical systems for the real-time analysis and sonification of movement qualities for persons with visual impairment (http://dance.dibris.unige.it/). As part of this project, sonification of two defined movement qualities was developed and evaluated in an interactive dance scenario with participants with and without visual impairment (Niewiadomski et al., 2019). Results showed that both qualities were recognized from sonification only, and the authors planned to extend their work to include more movement qualities. In order for sonification to facilitate the aesthetic experience of dance in the real world, it would have to go beyond the recognition of labeled expressive qualities, taking in consideration the non-declarative nature of the information conveyed in dance (Orgs et al., 2016; Stevens, 2017). Similar “silent disco” approaches as proposed for audio description studies could be applied for studies comparing different types of sonification, evaluating and comparing sighted and non-sighted participants' preferences. We would expect that, in addition to emotional qualities, coordination patterns would be a good focus for sonification, representing choreographic structures that involve spatial patterns as well as movement dynamics (Vicary et al., 2017). Sonification could refer to:

- the coordination and movement dynamics of body parts in a solo dancer;
- the coordination and interaction between two or three individual dancers;
- temporal coordination patterns within a group of dancers;
- spatial distributions of dancers and dynamic spatial structures.

Sonification of these factors would contain information about the entrainment of dancers with musical cues, with each other and within a group (Phillips-Silver et al., 2010; Clayton, 2012; Phillips-Silver and Keller, 2012), and might therefore support the listener's entrainment with the dancers, aiming at a kind of kinaesthetic empathy induced by auditive information.

### Third Field of Study: Multimodal and Interactive Choreography

Focusing on the aspect of entrainment addressed in section Neurocognitive perspectives in multimodal dance perception, it could be hypothesized that moving in (rhythmic) coordination with the dancers, at a close distance that allows to perceive breathing and other subtle body cues, has a positive effect on the audience members' emotional reaction and aesthetic evaluation of the dance performance. Going a step further toward multimodal choreography, a third line of research could therefore address the question to what extent dance performance should involve and integrate the audience to provide an appropriately immersive, enjoyable non-visual experience. This includes questions of spatial composition—where should the audience be placed in relation to the dancers? Should audience members stay in one place, or move, or be moved during the performance?—as well as questions of physical activity and interaction: should the audience be encouraged to move freely, if only on a small scale? Should the dancers teach them movement phrases before or during the performance to enable active participation? How should such movements be taught or modeled, via verbal instruction or explanation, haptic guidance, partnering? Some of these questions are neither easy to answer nor to study systematically as they touch issues of cultural and personal acceptance of physical contact and distance (a topic that has become even more sensitive during the covid-19 pandemic). While some audience members might feel inadequately exposed when being asked directly to dance with the dancers or learn dance steps (Kleege, 2014), a focus could be on the role of small-scale body movements in enhancing and intensifying the perception of dance. Movement marking—performing dance movements on reduced scale, or using gestures to represent full body movements—has shown to support dance learning as well as choreographic thinking (Kirsh, 2011; Warburton et al., 2013). We hypothesize that marking while perceiving a dance performance could not only enhance the later recall of the movements but also intensify the experience on an emotional level, through kinaesthetic engagement and enactment. While the sighted dance audience typical stays seated while watching the staged performance, encouraging movement even on a small scale could turn a multimodal dance performance into an active experience, thereby increasing its immersive character and aesthetic value.

According to artists' and scholars' views cited in previous sections, the non-visual experience of dance can be enhanced and intensified by the dancers' closeness that allows to perceive even subtle sounds and movement effects such as whispering, the rustling of clothes and movement of air, and by the audience members' own movement that becomes part of the performance. Bachrach et al. (2015) showed that attention toward the dancers' and one's own breathing and movement can enhance the audiences' entrainment with the dancers. Ideas and methodological approaches for integrating the audience spatially and prompting (inter-)actions can also be derived from the fields of dance improvisation (Blom and Chaplin, 1988; Østern, 2009), somatic practices (Eddy, 2009; Coogan, 2016) and immersive performance (Machon, 2013). In her essay on the work of Irish choreographer Joan Davis, Meehan (2017) states that contemporary immersive performance “can include audience involvement, sensual contact, opportunities for intimacy, direct encounter with space/objects/people and the inclusion of site or environment to stimulate engagement.” She proposes that this kind of work can “offer opportunities to reconsider social interactions with other people, the choices and judgements we make,” “and thereby” give food for thought rather than providing easy answers.” The latter could also be true for interactive, multimodal choreography that can be fully experienced without vision.

### Dancing Without Vision

Even though the main question of this article is how dance performance can be made accessible for a non-sighted audience, this last section will turn from dance perception toward active dancing, addressing insights from dance education and training for persons with visual impairment. Body awareness training and dance-based exercises were found to improve balance, gait, and basic motor skills in adults with acquired blindness (Larsson and Frändin, 2006). Arguing in favor of an inclusive dance education for young people with visual impairment, Seham and Yeo (2015) suggest that a combination of somatic practices, kinaesthetic approaches, tactile modeling, physical guidance, verbal description, and one-to-one partnering can support blind students in learning and developing novel movement concepts. These authors emphasize the potential of somatic training as it “de-emphasizes reliance on repeated visual cuing and brings mindful attention to internal sensory input,” helping students with visual impairment to “conceptualize and perceive dance through kinaesthetic sensorimotor experiences.” They also point toward the potential of verbal instructions and auditory imagery to optimize motor functioning in children with vision loss (Barati et al., 2014). Paxton et al. (1993) comment on the use of verbal explanation in their contact improvisation work with blind dancers: “by verbally telling someone how to move, we contact the intellectual mind. The mind can then ‘tell’ the body what to do, but the body does not understand words as well as it understands and remembers sensations and physical experience,” and continues: “verbal descriptions have to be metaphorical examples or analogies which employ language linked to the sense of touch and hearing, and occasionally taste and smell.” Echoing these words, neurocognitive research showed that individuals with visual impairment, including congenial blindness, can form concepts of movement and space based on multimodal sensations in much the same way as those without visual impairment (Ricciardi and Pietrini, 2011). There is neither reason to assume that people who cannot use vision therefore cannot dance, nor is there any good reason to believe than dance as an art form cannot be enjoyed by a non-sighted audience.

## Conclusions

Despite commonly adopted perspectives, dance does not have to be a primarily visual art from. As universal means of multimodal communication rooted deep in human evolution, dance links the visual, auditive, and haptic perception of other human bodies in motion to the perception of ones own body, movement and emotions. Like music, dance can convey non-declarative information relating to emotional states, yet drawing on the human brain's specific expertise for mapping perceived human motor action to related functions in one's own system. We suggest that the non-visual accessibility of dance performance should use two complementary channels. One time-coupled channel processes immediate, implicit information via auditive and bodily (haptic, proprioceptive, kinaesthetic, vestibular) cues, supporting entrainment with the dancers and immersion in the performance. To strengthen this channel, choreography can apply interactive formats that decrease the spatial distance between audiences and performers, facilitate direct contact between dancers and audience members, and allow the audience to move in coordination with the dancers. Integrating musical cues, movement sonification and specifically designed soundscapes can further support the immersive non-visual experience of dance performance. The other channel processes declarative information in the form of spoken language, typically applied via audio description. Its main purpose is to replace visual processing by filtering information and presenting pre-selected verbal content. As this channel is not directly time-locked with the performance, it has to be meticulously coordinated with the immediate one to blend in smoothly and provide for a fluent, holistic perceptive experience. Coordinating these two channels in a balanced way to elicit a pleasant, stimulating and immersive impression could be regarded as an overarching perspective in making dance a multimodal and potentially non-visual aesthetic experience to be enjoyed by sighted and non-sighted audiences.

## Data Availability Statement

Additional information about the case study is included in the article/Supplementary Material, further inquiries can be directed to the corresponding author/s.

## Author Contributions

A case report: Simon Mayer's “Sons of Sissy” is based on an unpublished essay written by EZ in 2016. Parts of Experiencing dance with all senses and Dance as a non-visual multi-sensory experience share material with a short book chapter in German language written by BB, published in 2020. All authors contributed to the article and approved the submitted version.

## Conflict of Interest

The authors declare that the research was conducted in the absence of any commercial or financial relationships that could be construed as a potential conflict of interest.
