# Supplementary material for: Dance Is More Than Meets the Eye—How Can Dance Performance Be Made Accessible for a Non-sighted Audience?
Source: Front Psychol. 2021 Apr 16;12:643848. doi: 10.3389/fpsyg.2021.643848 (PMC8085341; doi:10.3389/fpsyg.2021.643848)
Supplement: Supplementary file 1 [file Data_Sheet_1.pdf]

Supplementary material 1

Background information

"The Humane Body - Ways of Seeing Dance" is an EU project on which Wiener Tanzwochen (Vienna), Kaaitheater (Brussels), Center National de la Danse (Paris) and The Place (London) are working together (The Humane Body - Ways of Seeing Dance). The members strive to make contemporary dance accessible to a visually impaired audience by encouraging a change of direction in the way artists and organizations create and experience dance art. The aim is to create awareness of the underrepresented audience group of visually impaired people and to enable inclusion. Experts, artists and the visually impaired are currently working on this topic in workshops and symposia in order to further research the area. This includes, for example, the training of audiodescriptors and the execution of audio comments in different languages. In May 2016 the project initiated tours of Europe with the cooperating choreographers and their performances in order to strengthen the network and cooperation. Simon Mayer's *"Sons of Sissy"* is one of the participating performances, it was staged as part of the IMPULSTANZ festival, which takes place annually in Vienna (Impulstanz, 2016).

"Sons of Sissy" takes a new perspective on the tradition of folk customs, interpreting them in its own way, for example breaking with male role models or folk music (Wolf-Perez, 2015). Folk dance is ironically deconstructed in that "couple, turning and circle dances are reduced to their essentials" (Ploebst, 2016), as is the country costuming of the four dancers up to nudity. The dancers accompany themselves musically by yodelling and shoemaking, playing the violin, buttoned harmonica, double bass and horn, as well as whips and a big cow bell (Ploebst, 2016). The piece thrives on the ambivalence between folk customs and contemporary dance: at the beginning the colorfully dressed folk music quartet plays their music with a serious expression, then both dance and music break down into their individual pieces. Cheerful chunky music creates something like a mantra, the dancers walk rhythmically in a circle, accompanied by the drawn-out tones of the instruments. Then the movement is further broken down into extremes, the turning in folk dance is increased to the point of dizziness. The movements and music are reminiscent of "forms of ritual dance,

in which trance states can be achieved through constant repetition of movements and unchanging rhythms” (Krösche, 2015). After the four men have completely undressed, they return to the folk music quartet, then break out of it again by jumping wildly and moving like animals. Solos with improvised movements represent attempts to break free from the quartet, which, however, always comes back together (cf. Krösche, 2015). Two men also come closer to each other in a loving way.

The title “*Sons of Sissy*” alludes to the “kitsch brands Romy Schneider as and Kaiserin Sisi” (Ploebst, 2016), whereby the piece could be interpreted as follows: The sons, i.e. a new generation, questions rural tradition and wants to break away from it, which is expressed through the deconstrction of folk dance. Although this folk dance is constantly being modified, sometimes beyond recognition, it is nevertheless the starting point of the piece and remains omnipresent. Individual escapes from it play an important role: The liberation from the conventions, the ritualistic, can represent the liberation of the ego so that the human being is just its self: “naked body, [...] sweat, movement, breath and tense muscle" (Krösche, 2015).

References

Impulstanz (2016). Simon Mayer (BE/AT). Sons of Sissy. https://www.impulstanz.com/archive/performances/2016/id809/.

The Humane Body - Ways of Seeing Dance. http://www.thehumanebody.eu/

Krösche, K. (2015). Volkstanztrance. nachtkritik.de. https://nachtkritik.de/index.php?option=com_content&view=article&id=10795&catid=388&Itemid=100089

Ploebst, H. (2016). Dieser Tanz kickt Klischees vor sich her. Simon Mayers Erfolgsstück „Sons of Sissy“ gibt es jetzt auch mit Hörbeschreibung für Sehbehinderte. DER STANDARD. SPEZIAL IMPULSTANZ, (13.07.2016), 14.

Wolf Perez, E. (2015). Simon Mayer: Sons of Sissy. tanz.at. http://www.tanz.at/index.php/kritiken/kritiken-2015/1240-simon-mayer-sons-of-sissy
