# Supplementary material for: Dance Is More Than Meets the Eye—How Can Dance Performance Be Made Accessible for a Non-sighted Audience?
Source: Front Psychol. 2021 Apr 16;12:643848. doi: 10.3389/fpsyg.2021.643848 (PMC8085341; doi:10.3389/fpsyg.2021.643848)
Supplement: Supplementary file 2 [file Data_Sheet_2.pdf]

Supplementary material 2

The author's (EZ) personal perspective

In the following, I will describe how I (EZ) experienced the performance of "Sons of Sissy" at Vienna, 2016 (July 22nd) as an audience member. Even the beginning, it was very interesting for me as a sighted person to wear a blindfold while something happened on stage, wondering who sat next to me, and how to best take notes while not seeing anything. When the audio commentary started, I felt more supported and reassured: It was said that people came into the room, went up the stairs, and the performance would start in a few minutes. Sentences like "You are on the right frequency. Just for information, you have come to the right place" or even the cracking of the radio calmed me down when nothing was said for a longer time. Then the four dancers were described, their body size, facial features, stature, clothing, their musical instruments. It was also described where props such as incense bowl, tuba, whip and cow glove were located on the stage and how the lighting was used. In the following, the dancers were called by their first names; however, I found it difficult to assign the names to persons without remembering individual features from the description at the beginning.

Some of the audio comments were strange, I didn't know what to make of them: "M. begins to scratch the violin slightly to adjust the mood." As a listener, one perceives this change in musical sound and mood immediately, here the commentator should have either not given any comment and leave the interpretation to the listener or describe more explicitly in which way the mood was developing. Likewise, the sentence "The mood of the light changes" was too free, since "change" can be interpreted in any direction. Movement descriptions such as "spiraling steps", "turning in a row" and "jumping in improvised dance" were particularly difficult to interpret for me as a sighted person, and I imagined that this might cause even greater problems for a visually impaired person. I also had no idea what exactly was meant by "traditional dance". Such specific concepts of movements could be explained in more detail beforehand in the touch tour or even actively experienced by the participants themselves. The comment "They walk in a circle in lockstep" could easily be turned into a movement image on the basis of previous practice.

I also found the sentence "they are now repeating the sequence of the choreography in pairs" hard to understand, as it was not clear to me which part of the 60-minute choreography it referred to. “It feels like a liberation" also allowed too much space for interpretation. But there were also more explicit formulations such as "the lighting mood is dimmed and a circle with a dark cone in the middle appears on the floor”. Many descriptions referred to directions and locations such as “both trudge diagonally to the center of the stage and then back and out.” The expression of each individual dancer cannot be described at the same time, and just naming the trudge is insufficient for an idea of the scene. For example, facial expression and the gesture of the upper body play a role. Sometimes the piece becomes so loud, for example when the cowbells fall to the ground, that you can no longer hear the commentary. These moments are also very hectic, which I found quite exhausting, and would therefore leave such dramatic moments that have their own acoustic effect without comment. What is important for understanding the scene could be explained before or afterwards. When much of the scene was described, it was difficult for me to imagine this and perceive the music exactly at the same time. When the holy water was used, I perceived its smell and wondered if I, as a sighted spectator, would have perceived the smell at all.

I followed the second part of the performance partly sighted and partly with a blindfold. Now I saw that the people and the movements, which were sometimes really difficult to describe, were completely different from what I had imagined without vision. I also noticed that the comment usually started with a time lag, either before or after the event. This should be done consistently, in my opinion after the particular movement, so that the listener is initially given the chance to perceive the event exclusively through his or her own acoustics. When the performers were completely naked, the acoustic perception of their movement, for example clapping on the thighs, became more intense, but this part of the play also lived through visual perception. The comment “M. jumps the hardest” does not give any information about how intense and funny his jumping actually was, and the enormous expressivity of the actor cannot be adequately described by “jerky movement” either. There was no mention of the floor glistening with sweat. I also saw two actors getting extremely close, which the commentator described with “only a few centimeters between

the faces”; here I rather would have imagined “from a distance it looks like a kiss”. During another very hectic scene, I didn't know where to look because so much was happening on stage at the same time. In such moments, in my opinion, it is impossible for audio description to do justice to what is happening, since so many things cannot be described at once. As soon as the descriptor decides to focus on a person or situation, he or she neglects the others and thus sets an interpretation focus that is otherwise left to each observer individually. It might therefore be best if the comment paused.
